# Involvement of *hrpX* and *hrpG* in the Virulence of *Acidovorax citrulli* Strain Aac5, Causal Agent of Bacterial Fruit Blotch in Cucurbits

**DOI:** 10.3389/fmicb.2018.00507

**Published:** 2018-03-27

**Authors:** Xiaoxiao Zhang, Mei Zhao, Jianpei Yan, Linlin Yang, Yuwen Yang, Wei Guan, Ron Walcott, Tingchang Zhao

**Affiliations:** ^1^State Key Laboratory for Biology of Plant Diseases and Insect Pests, Institute of Plant Protection, Chinese Academy of Agricultural Sciences, Beijing, China; ^2^Department of Plant Pathology, University of Georgia, Athens, GA, United States; ^3^Faculty of Agronomy, Jilin Agricultural University, Changchun, China; ^4^Plant Protection College, Shenyang Agricultural University, Shenyang, China

**Keywords:** *hrpG*, *hrpX*, *Acidovorax citrulli*, type III secretion system, hypersensitive response, reactive oxygen species, pathogenicity, applications of biological

## Abstract

*Acidovorax citrulli* causes bacterial fruit blotch, a disease that poses a global threat to watermelon and melon production. Despite its economic importance, relatively little is known about the molecular mechanisms of pathogenicity and virulence of *A. citrulli*. Like other plant-pathogenic bacteria, *A. citrulli* relies on a type III secretion system (T3SS) for pathogenicity. On the basis of sequence and operon arrangement analyses, *A. citrulli* was found to have a class II *hrp* gene cluster similar to those of *Xanthomonas* and *Ralstonia* spp. In the class II *hrp* cluster, *hrpG* and *hrpX* play key roles in the regulation of T3SS effectors. However, little is known about the regulation of the T3SS in *A. citrulli*. This study aimed to investigate the roles of *hrpG* and *hrpX* in *A. citrulli* pathogenicity. We found that *hrpG* or *hrpX* deletion mutants of the *A. citrulli* group II strain Aac5 had reduced pathogenicity on watermelon seedlings, failed to induce a hypersensitive response in tobacco, and elicited higher levels of reactive oxygen species in *Nicotiana benthamiana* than the wild-type strain. Additionally, we demonstrated that HrpG activates HrpX in *A. citrulli*. Moreover, transcription and translation of the type 3-secreted effector (T3E) gene *Aac5_2166* were suppressed in *hrpG* and *hrpX* mutants. Notably, *hrpG* and *hrpX* appeared to modulate biofilm formation. These results suggest that *hrpG* and *hrpX* are essential for pathogenicity, regulation of T3Es, and biofilm formation in *A. citrulli*.

## Introduction

Bacterial fruit blotch (BFB), caused by the gram-negative bacterium *Acidovorax citrulli* (Schaad et al., 1978, 2008; Willems et al., 1992), is a globally occurring destructive disease of cucurbit crop species (Burdman and Walcott, 2012). Multiple studies have evaluated methods to mitigate the impact of BFB, including pathogen exclusion by quarantine, seed health testing, seed treatment, and field applications of biological control agents (Dutta et al., 2014; Jiang et al., 2015; Tian et al., 2016). Unfortunately, the success of these approaches has been limited, and BFB still poses a serious threat to commercial watermelon and melon production worldwide (Burdman and Walcott, 2012).

Despite the economic importance of BFB, little is known about the molecular mechanisms of *A. citrulli* pathogenesis (Burdman and Walcott, 2012). Recent studies have shown that important pathogenicity and virulence determinants of *A. citrulli* include the type II (T2SS) (Johnson, 2010), type III (T3SS) (Johnson et al., 2011), and type VI (T6SS) secretion systems (Tian et al., 2015); type IV pili (T4P) (Bahar et al., 2009a), polar flagella (Bahar et al., 2011), and quorum sensing (QS) (Wang T. et al., 2016). Most gram-negative, biotrophic, phytopathogenic bacteria rely on a functional T3SS to promote disease or trigger a hypersensitive response (HR) in susceptible and resistant plants, respectively (Büttner and Bonas, 2002). Importantly, gram-negative phytopathogenic bacteria secrete type 3-secreted effectors (T3Es) directly into plant host cells via the T3SS (Block et al., 2008; Van Engelenburg and Palmer, 2010; Nomura et al., 2011; Jiang et al., 2013; Xin et al., 2016). These T3Es may function to overcome pathogen-associated molecular pattern-triggered immunity (PTI) and effector-triggered immunity (ETI) (Cui et al., 2013) or promote effector-triggered susceptibility (Jehle et al., 2013). The genes encoding the components of the T3SS are named hypersensitive response and pathogenicity (*hrp*) genes (Kim et al., 2003; Tampakaki et al., 2010). The *hrp* genes are located in large clusters, generally 20–25 kb in size (Büttner and Bonas, 2002). On the basis of gene organization, sequence, and regulation, *hrp* clusters can be divided into two classes: class I contains clusters of *Pseudomonas syringae* and enteric plant-pathogenic bacteria, whereas class II contains the *hrp* genes of *Xanthomonas* spp. and *Ralstonia solanacearum* (Bogdanove et al., 1996; Büttner and Bonas, 2002). Analysis of the genome sequence of the *A. citrulli* group II strain AAC00-1 revealed the presence of a *hrp*-T3SS (Burdman and Walcott, 2012). Moreover, sequence and cluster organization analyses showed that the *A. citrulli hrp* cluster belongs to class II (Burdman and Walcott, 2012). The *hrp* genes are induced in plant leaves and in T3SS-inducing, nutrient-poor medium. Generally, T3SS is suppressed in rich medium (Schulte and Bonas, 1992; Clarke et al., 2010). Gram-negative bacterial plant pathogens of the genera *Erwinia, Pseudomonas, Ralstonia*, and *Xanthomonas* require a fully active T3SS for pathogenicity, and the recent establishment of an inducing medium has facilitated the study of *hrp* gene expression and regulation in *in-vitro* experiments (Yuan and He, 1996; Rossier et al., 1999; Murata et al., 2006; Ancona et al., 2015).

In *Xanthomonas* spp., the expression of *hrp* genes is controlled by two regulators: HrpG and HrpX. HrpG is an OmpR family regulator that activates the expression of *hrpX* in *X. campestris* pv. *vesicatoria* (Wengelnik et al., 1996b) and in *X. campestris* pv. *campestris* (Huang et al., 2009). HrpX, an AraC-type transcriptional activator, controls the expression of other *hrp* genes and some effector genes (Wengelnik et al., 1996a). The HrpG regulon regulates the expression of *hrp* genes, as shown by cDNA-amplified fragment length polymorphism analysis (Noël et al., 2001). Regulated genes include the *hrp* gene cluster, effector genes, and genes encoding proteases. Thirty HrpG-induced and five HrpG-repressed genes have been identified in *X. campestris* pv. *vesicatoria*. Phosphorylated HrpG is predicted to activate the expression of *hrpX* (Büttner and Bonas, 2010; Tampakaki et al., 2010). HrpX binds to a conserved *cis*-regulatory element called the plant-inducible promoter (PIP) (TTCGC-N15-TTCGC) or a less conserved PIP-like motif (TTCGC-N8-TTCGT) in promoter regions associated with *Xanthomonas* virulence (Fenselau and Bonas, 1995). However, the mechanism differs from that of the HrpL/HrpS-dependent systems in *Erwinia carotovora* and *Pseudomonas* spp., which belong to class I *hrp* clusters (Tampakaki et al., 2010). Recently, Guo et al. (2011) showed that *hrpG* and *hrpX* in *Xanthomonas axonopodis* pv. *citri* are involved in the regulation of multiple physiological functions, such as biofilm formation, by genome-wide microarray analyses. Thus, *hrp* gene regulation may be involved in a wide array of functions.

*A. citrulli* relies on a functional T3SS for pathogenicity (Ren et al., 2009; Burdman and Walcott, 2012; Eckshtain-Levi et al., 2014). Unfortunately, not much is known about the T3SS and T3Es in *A. citrulli* as compared to other plant-pathogenic bacteria (Burdman and Walcott, 2012) because of the lack of a T3SS-inducing medium for *A. citrulli* until recently (Chen, 2016). Recent research has focused mainly on the characterization of genetically distinct groups of *A. citrulli* strains and on host preference (Silva et al., 2016; Zivanovic and Walcott, 2017). In addition, the arsenal of T3S effectors in *A. citrulli* strains were reported to be related to host preference on melon fruits (Yan et al., 2017). More specifically, group I *A. citrulli* strains can cause water-soaked lesion on detached immature watermelon rinds, while group II strains cannot. Previously, we reported that the *A. citrulli* group II strain Aac5, which was isolated from watermelon in Taiwan also relies on the T3SS for pathogenicity. As expected, Aac5 lost the ability to infect watermelon when the homologous *hrcN* gene was deleted (Yan et al., 2015). In addition, biofilm formation was crucial for Aac5 virulence and may be related to the T3SS (Wang T. et al., 2016). Based on sequence analysis of *A. citrulli* strain AAC00-1, *hrpX* and *hrpG* homologs were identified. We hypothesized that the *hrpG* and *hrpX* homologs in *A. citrulli* strain Aac5 may function as T3SS regulators. Hence, the objective of this study was to characterize the roles of these homologs in *hrp* gene regulation and pathogenicity.

## Materials and methods

### Bacterial strains, plasmids, and growth conditions

The strains and plasmids used in this study are listed in Table 1. All *A. citrulli* strains were grown on King's B (KB) or T3SS-inducing medium (Chen, 2016) (10 g/L Bacto Peptone, 5 g/L yeast extract, 5 g/L NaCl, 10 mM MgCl_2_, pH 5.8, in sterilized distilled water [SDW]) at 28°C. *Escherichia coli* strains were grown on LB medium at 37°C (Sambrook and Russel, 2001). Liquid cultures of the strains were grown in sterilized test tubes containing KB, LB, or T3SS-inducing broth continuously agitated at 200 rpm on a rotary shaker (DDHZ-300; Taicang Experimental Instrument Factory, Jiangsu, China). When required, the growth media were supplemented with the following antibiotics: ampicillin (Amp), 100 μg/mL; chloromycetin, 3.4 μg/mL; and kanamycin (Km), 50 μg/mL. For assays, bacterial concentrations were estimated by optical density (OD_600_) using a spectrophotometer (Evolution 300 UV/VIS; Thermo Scientific, Waltham, MA, USA).

**Table 1:** Bacterial strains and plasmids used in this study.

**Strains and plasmids**	**Characteristics^a^**	**Reference**
***Acidovorax citrulli*** **STRAINS**		
Aac5	Wild-type group II strain; Amp^R^	Yan et al., 2013
ΔhrpG	*hrpG* markerless mutation of Aac5; Amp^R^	This study
ΔhrpX	*hrpX* markerless mutation of Aac5; Amp^R^	This study
ΔhrpG-comp	ΔhrpG containing pBBRNolac-4FLAG carrying *hrpG* with its native promoter; Amp^R^; Km^R^	This study
ΔhrpX-comp	ΔhrpX containing pBBRNolac-4FLAG carrying *hrpX* with its native promoter; Amp^R^; Km^R^	This study
WT-EV	Wild-type strain Aac5 transformed with pBBRNolac-4FLAG	This study
ΔhrpG-EV	ΔhrpG transformed with pBBRNolac-4FLAG	This study
ΔhrpX-EV	ΔhrpX transformed with pBBRNolac-4FLAG	This study
***Escherichia coli***		
*DH5α*	*supE44 ΔlacU169* (*Φ80/lacZ*ΔM15) *hsdR17 recA1 endA1 gyrA96 thi-1 relA1*	TIANGEN
***Pseudomonas syringae*** **STRAIN**		
DC3000	Wild-type strain; Rif^R^	Lab collection
Plasmids		
pK18mobsacB	Suicide vector with *sacB* gene; Km^R^	Kvitko and Collmer, 2011
pK18hrpG	Suicide vector containing upstream and downstream fragments of *hrpG* gene on pK18mobsacB; Km^R^	This study
pK18hrpX	Suicide vector containing upstream and downstream fragments of *hrpX* gene on pK18mobsacB; Km^R^	This study
pBBR1MCS-2	pBBR1MCS-2 containing pBluescript II KS-*lacZα*; Km^R^	Kovach et al., 1995
pBBRNolac-4FLAG	*lac* promoter was deleted from pBBR1MCS-2 and C-terminal 4 × FLAG tag was inserted; need native promoter to drive only.	This study
pBBR2166	pBBRNolac-4FLAG containing *Aac5_2166* gene with its native promoter	This study
pBBRNolacGUS	lac promoter was deleted from pBBR1MCS-2 and GUS reporter gene was inserted	This study
pBBRNolac2166GUS	pBBRNolacGUS containing *Aac5_2166* gene with its native promoter	This study
pBBRHBhrpG	pBBRNolac-4FLAG containing *hrpG* gene with its native promoter; Km^R^	This study
pBBRHBhrpX	pBBRNolac-4FLAG containing *hrpX* gene with its native promoter; Km^R^	This study
pRK600	Helper strain in tri-parental mating; Cm^R^	Lab collection
pYBA1132	plant expression vector containing 35S promoter, Km^R^	Lab collection
pYBA1132-2166	pYBA1132 containing *Aac5_2166* gene	This study
PJY-mini-TN7T-GUS	For cloning the GUS reporter, Amp^R^	Zhang et al., 2013

a *Amp^R^, Km^R^, and Rif^R^, and Cm^R^ indicate resistance to ampicillin, kanamycin, rifampicin, and chloramphenicol, respectively*.

### Molecular manipulations

Molecular manipulations were carried out using standard procedures (Sambrook and Russel, 2001). Constructs were ligated using ClonExpress II One Step Cloning Kit (Vazyme Biotech, Nanjing, China). Polymerase chain reaction (PCR) primers used in this study (Supplementary Table 1) were synthesized by BGI Laboratories (BGI, Shenzhen, China). KOD-Plus neo (Toyobo, Shanghai, China) and 2 × Taq Plus PCR MasterMix (Tiangen, Beijing, China) were used for PCR amplification.

### Construction of *hrpG* and *hrpX* gene deletion mutants and complementation

Markerless *hrpG* and *hrpX* mutants of *A. citrulli* strain Aac5 were generated through allele exchange (Ren et al., 2014). Primers were designed on the basis of *A. citrulli* AAC00-1 sequences for *Aave_0445* and *Aave_0444*, which encode *hrpG* and *hrpX* homologs, respectively, and their flanking regions. The flanking regions were cloned from Aac5 and were aligned to those of to AAC00-1 using DNAMAN version 5.2.2 (Lynnon Biosoft, Quebec, Canada), and the sequence similarity was 100%. For the *hrpG* gene (*Aave_0445*), the upstream and downstream flanking regions were amplified by PCR using the primer pairs hrpG-1F/hrpG-1R and hrpG-2F/hrpG-2R (Supplementary Table 1), respectively. The two fragments were concatenated by overlap PCR (Zhang et al., 2013) and subsequently ligated into the suicide plasmid, pk18mobsacB, to generate pk18hrpG. pk18hrpG was transformed into *A. citrulli* strain Aac5 by tri-parental mating with an *E. coli* strain carrying the helper plasmid, pRK600. Single crossover colonies were selected on KB plates containing Amp and Km. Individual transformants were then sub-cultured continuously in KB liquid medium containing Amp alone to promote double crossover homologous recombination. The *hrpG* deletion mutant, ΔhrpG, which was resistant to Amp but sensitive to Km, was confirmed by PCR using the primer pairs hrpG-TF/hrpG-TR, hrpG-1F/hrp-2R, km-F/km-R, and WFB1/WFB2 (Supplementary Table 1).

To construct a complementation vector, the complete *hrpG* gene (1,074 bp) including its native promoter, was amplified with primers HBhrpG-F/HBhrpG-R (Supplementary Table 1) and inserted into the shuttle vector, pBBRNolac-4FLAG, to create pBBRHBhrpG. The pBBRNolac-4FLAG was constructed by deleting the lac promoter of pBBR1MCS-2 using a KOD-Plus-Mutagenesis Kit (Toyobo, Shanghai, China), and inserting a 4 × FLAG tag into the vector with C-terminal fusion expression, so that only the native promoter drove target gene expression. The complementation vector pBBRHBhrpG was introduced into the ΔhrpG mutant to generate the complemented strain ΔhrpG-comp. HrpG expression was verified in the complemented strain by western blotting (Sambrook and Russel, 2001). Similar methods were used to generate ΔhrpX and ΔhrpX-comp mutant strains from *A. citrulli* Aac5.

### Roles of *hrpG* and *hrpX* in *A. citrulli* virulence and hypersensitive response induction

To determine the roles of *hrpG* and *hrpX* in seed-to-seedling BFB transmission, seed transmission assays were performed as described previously (Bahar et al., 2009b), with slight modifications. The WT, ΔhrpG, and ΔhrpX Aac5 strains, were cultured in liquid KB medium and adjusted to 5 × 10^6^ CFU/mL with sterilized distilled water. Watermelon seeds (cv. Ruihong, *n* = 24) were soaked in a bacterial cell suspension (~5 × 10^6^ colony-forming units [CFU]/mL) at room temperature for 2 h with continuous agitation at 60 rpm using a rotary shaker (Ecotron; Infors HT, Bottmingen, Switzerland). The inoculated seeds were then air-dried for 24 h and sown in plastic pots (Rongxiangruihe, Beijing, China, six seeds per pot) filled with a potting mix (vermiculite, soil, and peat at a 1:1:1 ratio). The pots were placed in a growth chamber for 12 days under the following conditions: 25–30°C; 65% mean relative humidity, and 16 h light/8 h darkness cycle during the experiments. BFB severity was visually assessed 12 days after seedling emergence, as described previously (Bahar et al., 2009b).

To determine the roles of *hrpG* and *hrpX* in *A. citrulli* virulence on watermelon seedlings, infiltration assays were performed as described previously (Ren et al., 2014). Briefly, cotyledons of 2-week-old watermelon seedlings (cv. Ruihong, *n* = 6) were syringe-infiltrated with bacterial suspensions (2 × 10^4^ CFU/mL) and incubated at room temperature. Cotyledons were photographed at 24, 48, 72, and 96 h post inoculation (hpi) using an EOS 70D camera (Canon, Beijing, China). As a negative control, plants were injected with 10 mM MgCl_2_.

To evaluate the effects of *hrpG* and *hrpX* deletion on HR induction, HR assays were performed by injecting a bacterial cell suspensions into the leaves of 3-week-old tobacco (*Nicotiana tabacum*), as described previously (Ren et al., 2014). Leaves were visually examined for HR (tissue collapse) 24 h after infiltration. Electrolyte leakage was quantified for detecting HR on 3-week-old tobacco leaves injected with wild-type *A. citrulli* Aac5, ΔhrpG, and ΔhrpX, and the respective complementation strains, as described previously (Stork et al., 2015). In brief, the strains were inoculated into tobacco leaves, and four leaf disks from each treatment were harvested after 24 h, washed, and floated on 5 ml of distilled water for 4 h with gentle shaking prior to measuring the conductivity of the bath water. As a negative control (mock treatment), 10 mM MgCl_2_ was used.

### Assay for reactive oxygen species (ROS)

ROS were measured by chemiluminescence as described previously (Liu et al., 2015), with slight modifications. *N. benthamiana* leaves were inoculated with cell suspensions of *A. citrulli* Aac5, ΔhrpG, and ΔhrpX, and respective complementation strains (~10^8^ CFU/mL). *P. syringae* pv. *tomato* DC3000 was used as a negative control. At 24 hpi, leaf disks (0.4 cm in diameter) were excised and placed into wells of 96-well plates pre-loaded with 100 μL SDW. Subsequently, 100 μL of 0.5 mM L-012 (Wako, Guangzhou, China) in 10 mM MOPS-KOH buffer (pH 7.4) were added to each well. The intensity of ROS generation was determined as described by Liu et al. (2015). *N. benthamiana* leaf disks infiltrated with *P. syringae* pv. *tomato* DC3000 were used as a negative control.

### Evaluation of biofilm formation

To determine the roles of *hrpG* and *hrpX* in biofilm formation, assays were performed using *A. citrulli* wild-type Aac5, ΔhrpG, and ΔhrpX, and respective complementation strains, as previously described (Bahar et al., 2009a), with slight modifications. Briefly, 24-well plates (Corning, NY, USA) were pre-loaded with liquid T3SS-inducing medium and inoculated with a 1:1,000 dilution of overnight cultures of *A. citrulli* Aac5, ΔhrpG, and ΔhrpX, and the respective complementation strains. The plates were incubated in a tilted position at 28°C for 48 h, without agitation. Then, 0.1% crystal violet was added to each well for 30 min, after which the wells were washed with distilled water. Biofilm formation for each strain was compared quantitatively by solubilizing the stained biofilms with 100% ethanol and measuring the OD_590_ of the stained-cell suspensions with a spectrophotometer.

### Quantitative reverse-transcription (qRT-)PCR

Total RNA was extracted from wild-type *A. citrulli* Aac5, ΔhrpG, and ΔhrpX cultured in T3SS-induced liquid medium up to OD_600_ = 0.45, using TRIzol reagent (Invitrogen, Waltham, MA, USA). Contaminant DNA was digested and cDNA was synthesized using ReverTra Ace qPCR RT MasterMix with gDNA Remover (Toyobo, Shanghai, China). The mRNA levels were quantified by qPCR using KOD SYBR qPCR Mix (Toyobo, Shanghai, China) on an Applied Biosystems 7500 instrument (ABI, Waltham, MA, USA). The primers used for qRT-PCR are listed in Supplementary Table 1. Each sample was assayed in triplicate. *rpoB* was used as a reference gene. Relative expression of genes of interest was calculated by the 2^−ΔΔ*CT*^ method (Livak and Schmittgen, 2001).

### β-glucuronidase (GUS) reporter activity assay for detecting promoter activity

To construct a GUS reporter vector, the *GUS* gene was inserted into PBBRNolac (without 4 × FLAG-tag) to generate the PBBRNolacGUS vector using restriction enzymes *Xba*I and *Hind*III. The native promoter sequence of the putative T3S effector *Aac5_2166* (GenBank accession MG879253) was cloned from genomic DNA of *A. citrulli* Aac5 by PCR, and the product was ligated into PBBRNolacGUS to generate pBBR2166GUS. pBBR2166GUS and PBBRNolacGUS (negative control vector) were transformed into Aac5, ΔhrpG, and ΔhrpX to generate WT-2166GUS, hrpG-2166GUS, hrpX-2166GUS, and WT-GUS strains (negative control strain). Promoter activity was detected as previously described (Zhang et al., 2013).

### Assay of HrpG and HrpX protein production by *A. citrulli* strains in T3SS-inducing media

HrpX and HrpG protein expression was quantified by western blotting. *A. citrulli* strains (OD_600_ = 1.0) were pre-incubated for ~12–18 h in KB broth and washed twice. Twenty microliters of the bacterial suspension were transferred into 10 mL of T3SS-inducing medium and incubated at 28°C until the OD_600_ reached 0.5. To extract intracellular protein, 4 mL of the bacterial suspension as cell lysate was treated with Protease Inhibitor Cocktail (Bimake, Shanghai, China). The lysate was centrifuged at 12,000 × *g* and 4°C for 3 min, and the cells were resuspended in 200 μL of 4 × Laemmli Sample Buffer (Bio-Rad, Beijing, China) and heated for 10 min.

To extract secreted protein, the bacterial cell supernatants were treated with Protease Inhibitor Cocktail (Bimake) and separated by centrifugation, as previously described (Liu et al., 2017). Proteins were precipitated with 10% trichloroacetic acid (Sigma, Shanghai, China), separated by sodium dodecyl sulfate-polyacrylamide gel electrophoresis on 12.5% gels, and transferred to PVDF membranes (Millipore, Shanghai, China). The PVDF membranes were blocked with Tris-buffered saline plus Tween-20 (Sigma, Shanghai, China) containing 5% skim milk powder for 1 h at room temperature. After incubation with primary and secondary antibodies, the PVDF membranes were used for protein detection with Immobilon Western Chemiluminescent HRP Substrate (Millipore, Shanghai, China) by LAS 4000 (GE Biotech, Marlborough, MA, USA). The sources and dilutions of antibodies were as follows: anti-FLAG antibodies (anti-DDDDK-tag mAb-HRP-DirecT; 1:2,500 dilution; MBL, Beijing, China), anti-HrpG antibodies (1:1,000 dilution; Abclonal, Wuhan, China), anti-HrpX antibodies (1:1,000 dilution; Abclonal), and anti-glyceraldehyde 3-phosphate dehydrogenase (GAPDH) antibodies (1:1,000 dilution; Abclonal). The secondary antibodies were anti-rabbit antibodies (MBL) used at a 1:5,000 dilution. Protein quantification was carried out using ImageJ software (NIH, Bethesda, MD).

### Statistical analysis

All experiments were conducted three times. Six biological replicates were used in each biofilm assay, electrolyte leakage quantification assay, and promoter activity. Twelve biological replicates were used for ROS detection. Twenty-four leaves as biological replicates were used for assessing BFB severity. For the other assays, three biological replicates were evaluated. Data were analyzed by one-way analysis of variance (ANOVA) and Tukey's honest significant difference (HSD) tests. For qRT-PCR, data were analyzed by the independent-samples *t*-test. Statistical analyses were conducted using SPSS version 17.0 (SPSS, Chicago, IL, USA) and GraphPad PRISM 5.0 software (GraphPad Software, La Jolla, CA, USA). Differences with *p* < 0.05 were considered significant.

## Results

### Sequence analysis of HrpG and HrpX homologs in *A. citrulli* strain Aac5

**Figure 1:**
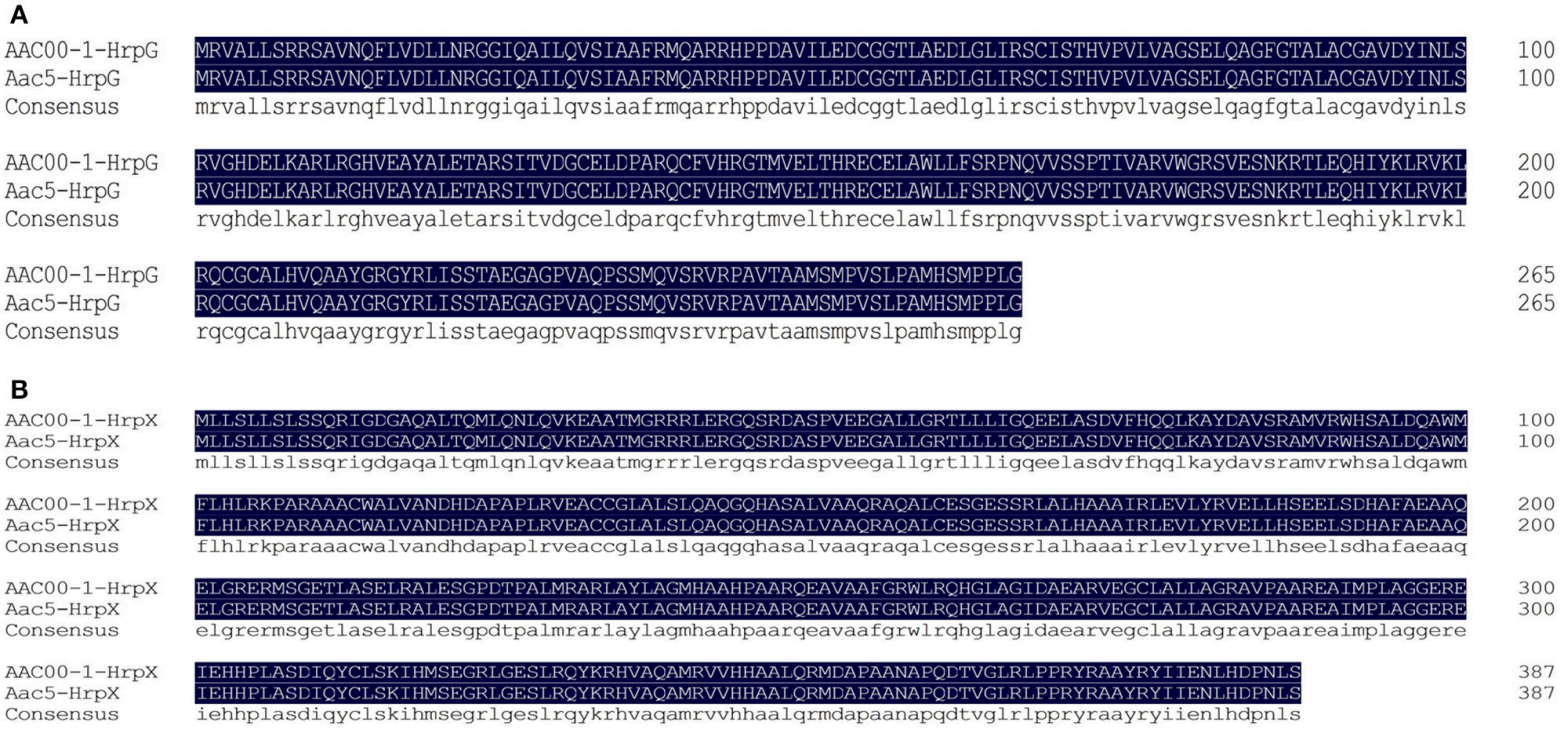
Multiple alignments of the deduced amino acid sequences of HrpG and HrpX proteins. **(A)** HrpG with AAC00-1 and Aac5; **(B)** HrpX with AAC00-1 and Aac5.

The group II *A. citrulli* strain Aac5 has been routinely used in studies on pathogenicity (Wang T. et al., 2016). BLAST searches using the AAC00-1 genome (GenBank accession NC_008752) identified *Aave_0445* and *Aave_0444*, which encode response regulator receiver protein (A1TJB4), which showed 100% amino acid sequence identity with HrpG (Q5EF45), and transcriptional regulator, AraC family (A1TJB3), which showed 100% amino acid sequence identity with HrpX (Q5EF44) in UniProt, respectively. The predicted HrpG (Q5EF45) and HrpX (Q5EF44) in UniProt were also from *A. citrulli*. To verify whether homologous genes coding HrpG and HrpX exist in strain Aac5, we cloned the sequences from Aac5 and found that they were 100% identical to the sequences of *Aave_0445* and *Aave_0444* from AAC00-1, named *hrpG* (GenBank accession no.: MG879251) and *hrpX* (MG879252), respectively. Amino acid sequence alignment to AAC00-1 using DNAMAN version 5.2.2 (Figures 1A,B) revealed 100% identity. PCR with the primer pair hrpG-TF/hrpG-TR yielded a 417-bp amplicon when using DNA template from wild-type Aac5, but not when the DNA was from the ΔhrpG mutant. DNA from wild-type Aac5 and ΔhrpG yielded 2,249- and 3,027-bp PCR amplicons, respectively, with the primer pair hrpG-1F/hrp-2R, and no amplicons were generated with the primer pair km-F/km-R. To confirm the identity of the wild-type Aac5 and ΔhrpG mutant, a 360-bp amplicon was produced with the diagnostic primer pair WFB1/WFB2. The *hrpX* gene deletion mutant was confirmed using the same approach.

### Roles of hrpG and hrpX in *A. citrulli* biofilm formation

**Figure 2:**
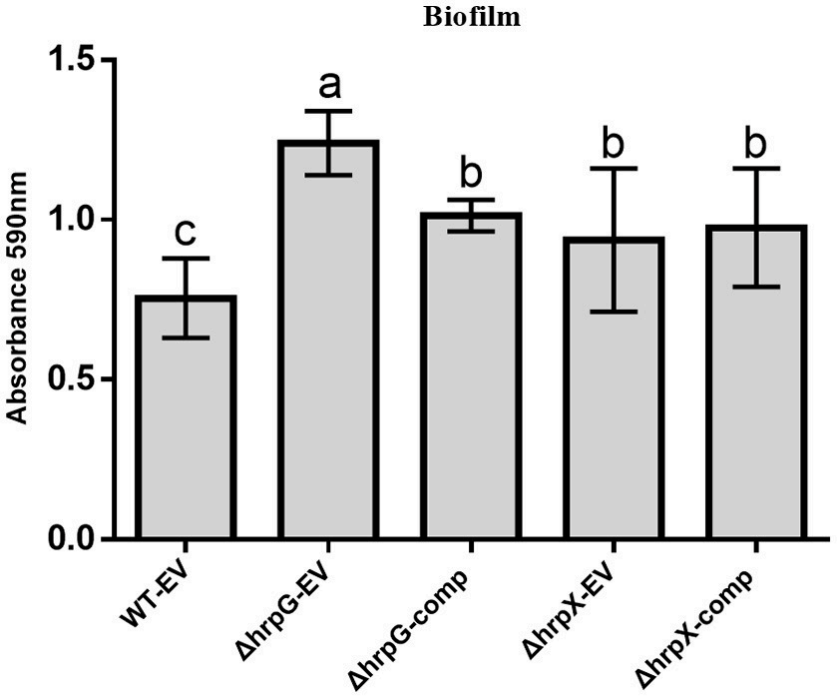
Effects of *hrpG* and *hrpX* genes on *Acidovorax citrulli* biofilm formation. The biofilm formation of *A. citrulli* in 24-well multi-dishes as quantified using crystal violet staining. WT-EV, wild-type strain Aac5 transformed with empty vector, pBBRNolac-4FLAG; ΔhrpG-EV, *hrpG* mutant transformed with empty vector, pBBRNolac-4FLAG; ΔhrpX-EV, *hrpX* mutant transformed with the empty vector pBBRNolac-4FLAG; ΔhrpG-comp, *hrpG* complemented with vector, pBBRHBhrpG (native promoter); ΔhrpX-comp, *hrpX* complemented with the vector, pBBRHBhrpX (native promoter). Each column shows the mean and standard deviation. The experiment was conducted three times. Averages and standard deviations from one of three experiments with similar results are shown. Different letters above bars indicate statistically significant differences as determined by one-way analysis of variance (ANOVA) and Tukey's honest significant difference (HSD), *p* < 0.05.

Since biofilm formation is involved in *A. citrulli* virulence and pathogenicity (Bahar et al., 2009a; Tian et al., 2015; Wang T. et al., 2016), we investigated the roles of *hrpG* and *hrpX* in biofilm formation. To rule out interference by plasmid introduction, we additionally transformed the empty vector, pBBRNolac-4FLAG, into wild-type *A. citrulli* Aac5 (WT-EV), ΔhrpG (ΔhrpG-EV), and ΔhrpX (ΔhrpX-EV). Biofilm formation was quantified by crystal violet staining, which showed that ΔhrpG-EV and ΔhrpX-EV exhibited significantly enhanced biofilm formation as compared with Aac5. The ΔhrpG-comp strain produced significantly less biofilm than ΔhrpX-EV, but significantly more than the wild-type strain (Figure 2). ΔhrpX-comp produced significantly more biofilm than the wild-type strain.

### *hrpG* and *hrpX* mutants of *A. citrulli* fail to induce HR in *N. tabacum*

**Figure 3:**
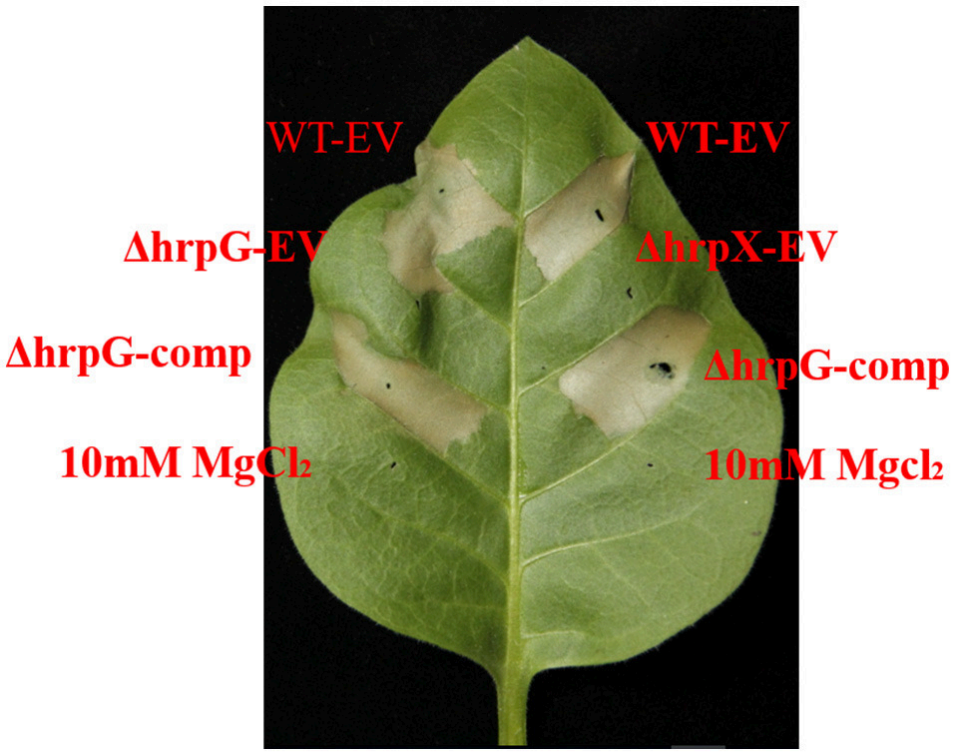
Effects of *hrpG* and *hrpX* gene on *Acidovorax citrulli* inducing a hypersensitive response (HR) on *Nicotiana tabacum*. Loss of HR-inducing ability on *N. tabacum* by the *hrpG* and *hrpX* mutants. The wild-type Aac5 and mutant carrying empty pBBRNolac-4FLAG and complemented strains ΔhrpG-comp, ΔhrpX-comp were inoculated into the *N. tabacum* leaves at a concentration of OD_600_ = 0.3 and incubated for 24 h. Ten millimolars of MgCl_2_ was used as a negative control. The photographs were obtained using EOS 70D (Canon, Japan). The experiment was conducted three times.

To determine whether *hrpG* and *hrpX* are involved in HR induction by *A. citrulli*, we examined HR induction by the mutant and wild-type strains in *N. tabacum*, a non-host plant typically used to test HR induction (Ren et al., 2009). The strains ΔhrpG-comp and ΔhrpX-comp induced an HR in *N. tabacum* similar to that induced by the WT-EV strain after 24 h. In contrast, ΔhrpG-EV and ΔhrpX-EV did not elicit an HR (Figure 3). These observations suggested that Aac5 requires *hrpG* and *hrpX* to induce an HR in *N. tabacum*.

**Figure 4:**
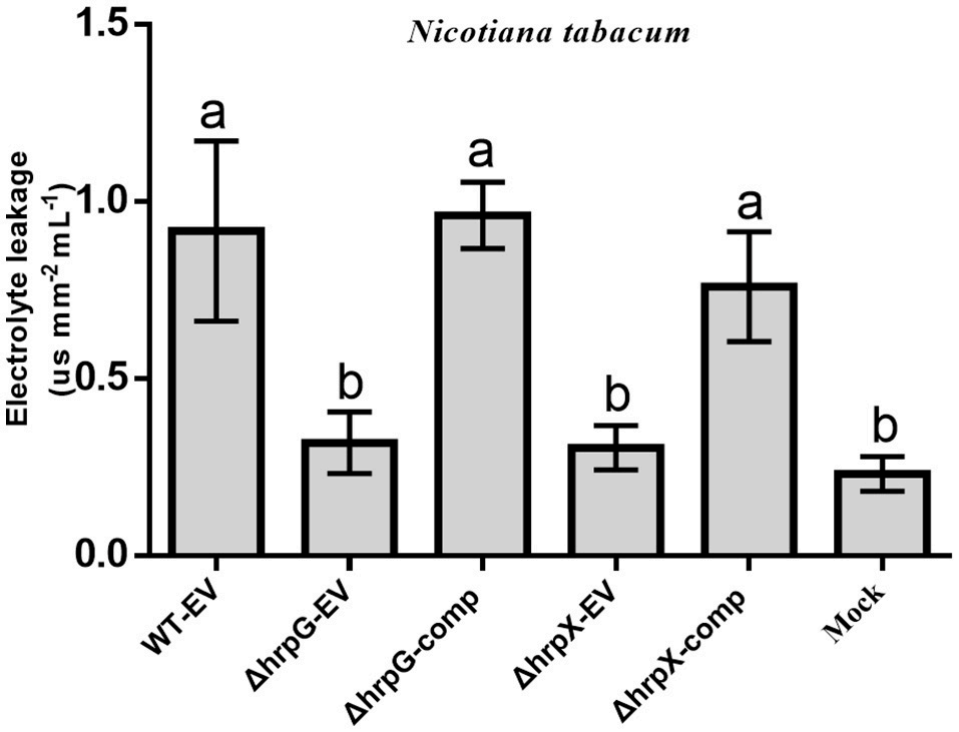
Electrolyte leakage quantification assay for verification of hypersensitive response (HR) in *Nicotiana tabacum* induced by *Acidovorax citrulli* strains. Electrolyte leakage quantification for the detection of HR. Values are mean conductivity of bathing water (μS mm^−2^ leaf ml^−1^ bath water) ± standard deviation (SD) (*n* = 3 plants). Different letters above bars indicate statistically significant differences as determined by one-way analysis of variance (ANOVA) and Tukey's honest significant difference (HSD), *p* < 0.05.

To substantiate these results, we used electrolyte leakage analysis. As expected, ΔhrpG-comp and ΔhrpX-comp induced electrolyte leakage to levels similar to those induced by WT-EV. In contrast, ΔhrpG-EV and ΔhrpX-EV induced significantly lower levels of electrolyte leakage, similar to that induced by mock treatment and 3-fold lower than the level induced by WT-EV (Figure 4). Overall, these findings revealed that Aac5 requires *hrpG* and *hrpX* to induce an HR in *N. tabacum*.

### *hrpG* and *hrpX* negatively regulate PTI-associated ROS generation

**Figure 5:**
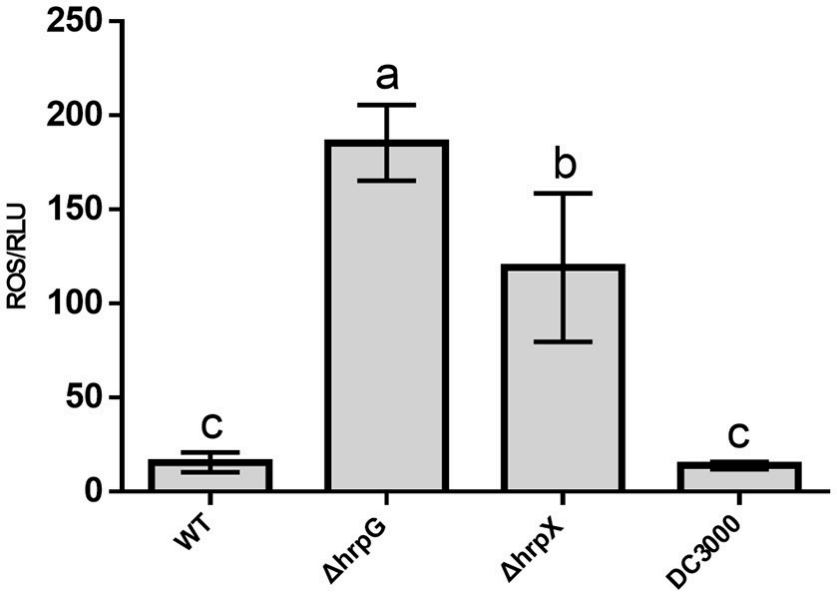
Effects of *hrpG* and *hrpX* gene on *Acidovorax citrulli* inducing ROS on *Nicotiana benthamiana*. Reactive oxygen species (ROS) generation by the wild-type *A. citrulli* strain Aac5 and the *hrpG and hrpX* deletion mutants. ROS assay results are presented as mean and standard deviations based on three replicates from three different plants. Different letters above bars indicate statistically significant differences as determined by one-way analysis of variance (ANOVA) and Tukey's honest significant difference (HSD), *p* < 0.05.

As *hrpG* and *hrpX* were found to be essential for HR induction by *A. citrulli*, we investigated whether PTI-associated ROS was altered during the *A. citrulli*–host interaction. Thus, we examined the ROS levels induced by the *A. citrulli* strains in *N. benthamiana* leaves using chemiluminescence (Liu et al., 2015). ΔhrpG and ΔhrpX elicited significantly higher levels of ROS (~12-fold and ~8-fold, respectively) than the WT strain, which induced ROS to a level similar to that of DC3000 (Figure 5). These results indicated that *hrpG* and *hrpX* act as negative regulators of PTI-associated ROS generation in the interplay between *A. citrulli* and *N. benthamiana*.

### *hrpG* and *hrpX* contribute to *A. citrulli* virulence on watermelon seedlings

**Figure 6:**
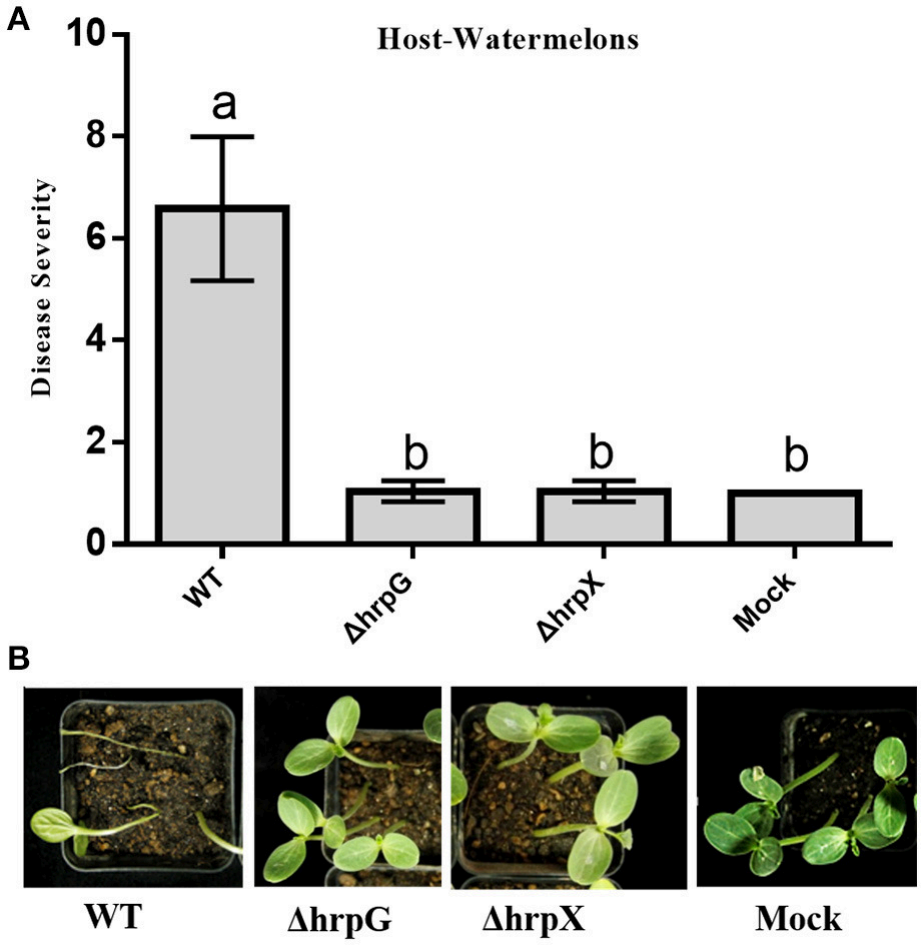
Contributions of *hrpG* and *hrpX* gene to *Acidovorax citrulli* virulence. Bacterial fruit blotch severity on watermelon induced by wildtype (Aac5), *hrpG* and *hrpX* deletion mutants of *A. citrulli* in seedling transmission assays. Disease severity was assessed according to (Bahar et al., 2009b). The experiment was conducted three times. **(A)** Disease severity of plants inoculated with different strains. The 10 mM MgCl_2_ was used as a mock treatment. Each column shows the mean and standard deviation. Different letters above bars indicate statistically significant differences as determined by one-way analysis of variance (ANOVA) and Tukey's honest significant difference (HSD), *p* < 0.05; **(B)** representative picture was taken after 12 days by EOS 70D (Canon, Japan).

Based on the above observations, we hypothesized that *hrpG* and *hrpX* are critical for *A. citrulli* virulence. We tested this hypothesis by conducting seed transmission and seedling infiltration assays in watermelon. The mean BFB severity index upon inoculation of ΔhrpG and ΔhrpX was similar to that of mock treatment, but ~7-fold lower than that upon inoculation of the WT strain (Figure 6A). Figure 6B shows that the WT strain caused more severe BFB symptoms than the ΔhrpG and ΔhrpX mutants. In the tissue infiltration assays, we observed that ΔhrpG and ΔhrpX did not cause BFB symptoms on watermelon cotyledons between 24 and 96 hpi. In contrast, the WT and complemented strains elicited BFB symptoms by 36 hpi (Supplementary Images 1A,B). Overall, these results showed that *hrpG* and *hrpX* contribute to *A. citrulli* virulence on watermelon seedlings.

### Interaction between HrpG and HrpX

**Figure 7:**
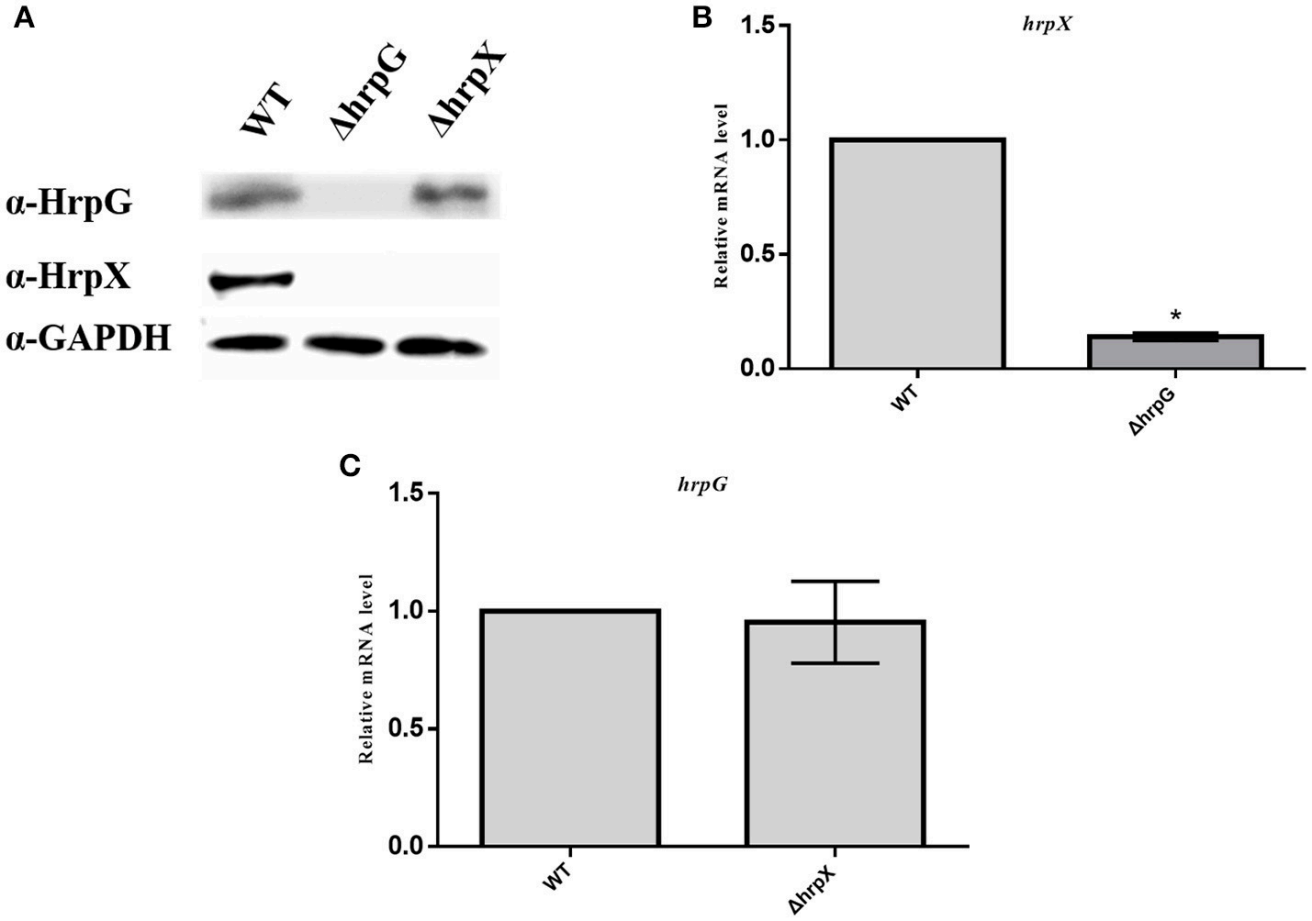
The interaction of *hrpG* and *hrpX* in *Acidovorax citrulli*. **(A)** Western blotting analysis showed that HrpG activated HrpX. The WT, ΔhrpG, and ΔhrpX strains were cultured in liquid T3SS-inducing medium up to OD_600_ = 0.5. Total cell extracts were analyzed by SDS-PAGE and immunoblotting, using specific antibodies. The experiment was performed three times. **(B)** Represents real-time quantitative RT-PCR to determine *hrpX* mRNA levels. *hrpX* expression was analyzed through RT-qPCR in wild-type strains Aac5 and the *hrpG* mutant strain, using specific primers. Bacteria were grown in liquid T3SS-inducing medium to OD_600_ = 0.45 and harvested to extract total RNA. **(C)** Represents real-time quantitative RT-PCR to determine *hrpG* mRNA levels. *hrpX* expression was assayed through RT-qPCR in wild-type strain Aac5 and the *hrpX* mutant, using specific primers. All the data shown are means ± standard deviation of duplicate samples from one representative experiment and are reported as fold induction relative to expression of WT. The *rpoB* gene was used as a reference gene. Each column shows the mean and standard deviation. The experiment was performed three times, with similar results obtained each time. Asterisks above bars indicate significant differences as determined by *t*-test, *p* < 0.05.

To evaluate the interaction between HrpG and HrpX, we assessed their translation levels by western blot analysis using anti-HrpX and anti-HrpG antibodies. Total protein was extracted from Aac5, ΔhrpG, and ΔhrpX cultured in T3SS-inducing medium. As shown in Figure 7A, HrpX expression was not detected in ΔhrpG when compared to the wild-type strain (as expected, HrpX was not detected for ΔhrpX), while HrpG expression in ΔhrpX was similar to the WT strain (as expected, HrpG was not detected for ΔhrpG). These findings indicated that HrpG might be the upstream regulator of HrpX in *A. citrulli*.

qRT-PCR revealed that the HrpX transcript levels in the *hrpG* mutant were ~7-fold lower than those in the WT strain (Figure 7B), whereas *hrpG* transcript levels were not affected in the *hrpX* mutant as compared to the WT strain (Figure 7C). These results indicated that *hrpG* regulates the expression of *hrpX*, but *hrpX* does not regulate the expression of *hrpG*.

### *hrpG* and *hrpX* regulate the expression of T3Es

**Figure 8:**
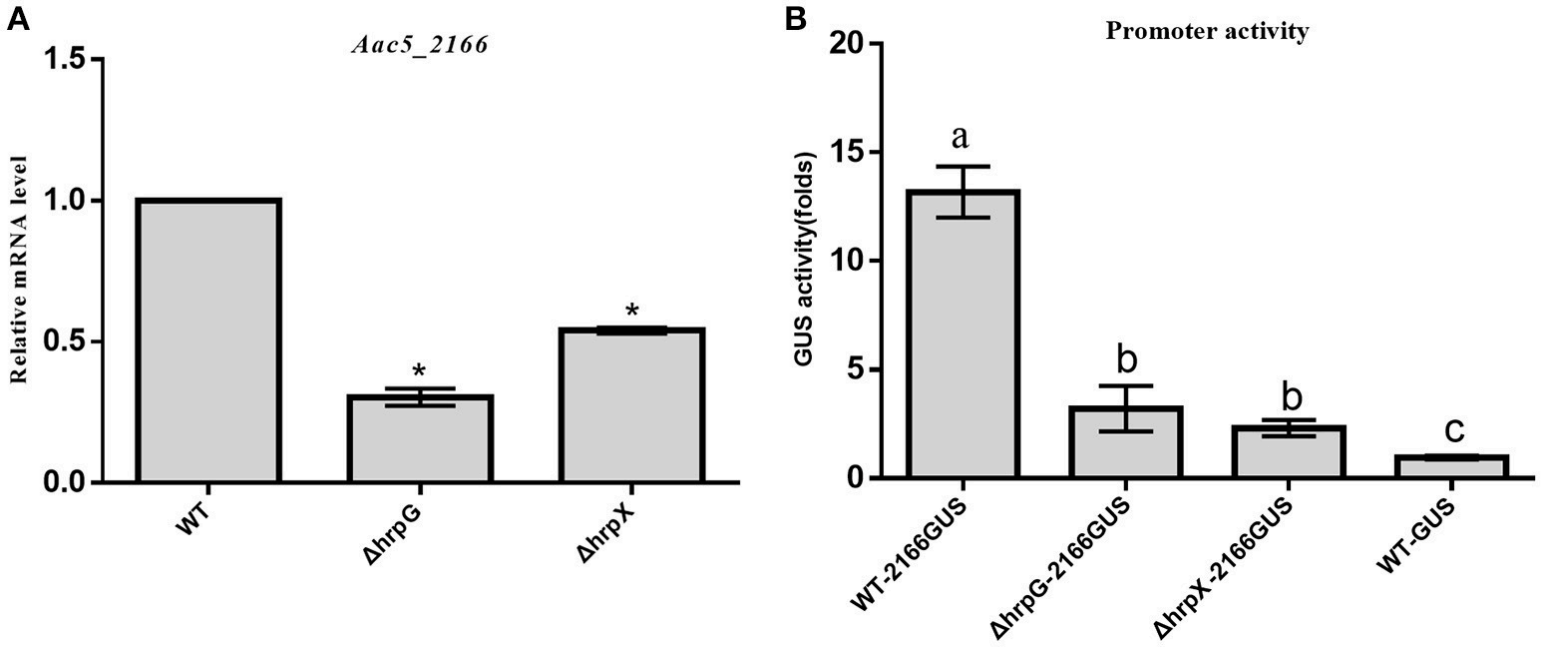
Effects of *hrpG* and *hrpX* genes on the regulation of type 3 effectors in *Acidovorax citrulli*. **(A)** Real-time quantitative RT-PCR was used to determine *Aac5_2166* mRNA levels. Putative type 3 secreted effector (T3E) *Aac5_2166* gene expression was assayed through RT-qPCR in wild-type strain Aac5 and the *hrpG* and *hrpX* deletion mutant strains, using specific primers. The data shown are means ±standard deviation of duplicate samples from one representative experiment and are reported as fold induction relative to expression of WT. The *rpoB* gene was used as a reference gene. The experiment was performed three times, with similar results obtained each time. Asterisks above bars indicate significant differences as determined by *t*-test, *p* < 0.05; **(B)** The detection of promoter activity of the putative T3E *Aac5_2166* was determined by assaying GUS activity. The pBBR2166GUS was constructed with *Aac5_2166* native promoter and GUS reporter gene. The wild-type strain Aac5 transformed with vector pBBR2166GUS carrying the GUS reporter gene, WT-2166GUS; the *hrpG* mutant transformed with vector pBBR2166GUS carrying the GUS reporter gene, ΔhrpG-2166GUS; the hrpG mutant transformed with vector pBBR2166GUS carry GUS reporter gene, ΔhrpX-2166GUS; the wild-type strain Aac5 transformed with pBBRNolacGUS without promoter sequence, WT-GUS (negative control). Each column shows the mean and standard deviation. All experiments were performed three times, with similar results obtained each time. Different letters above bars indicate significant differences as determined by one-way analysis of variance (ANOVA) and Tukey's honest significant differences test, *p* < 0.05.

Based on the above results, we hypothesized that the expression of T3Es in *A. citrulli* is regulated by HrpG and HrpX. To test this hypothesis, we used qRT-PCR to compare the transcript levels of T3Es in ΔhrpG, ΔhrpX, and the WT strain in T3SS-inducing medium. We transiently expressed *Aave_2166* homolog, encoding the YopJ homolog (Eckshtain-Levi et al., 2014; Traore, 2014), as a putative effector inducing programmed cell death in *N. benthamiana* (Supplementary Image 2) in WT, ΔhrpG, and ΔhrpX. Since the gene cloned from Aac5 has 100% sequence identity with the sequence of *Aave_2166*, it was named *Aac5_2166* (GenBank accession MG879253). We then measured the transcript levels of *Aac5_2166* in ΔhrpG, ΔhrpX, and the WT strain. The mRNA level of *Aac5_2166* was significantly lower in ΔhrpG and ΔhrpX (~3-fold and ~2-fold, respectively) than in the WT strain. These results indicated that *hrpG* and *hrpX* regulate the expression of *Aac5_2166* (Figure 8A).

To verify these results, we analyzed the promoter activity of *Aac5_2166*. We constructed a reporter vector, pBBRNolacGUS to measure promoter activity. In pBBRNolacGUS, the constitutive lac promoter was deleted and the *GUS* reporter gene was inserted, which is commonly used to analyze promoter activity in bacteria (Zhang et al., 2013; Wang L. et al., 2016). The results showed that the promoter activities of *Aac5_2166* in the ΔhrpG-2166GUS and ΔhrpX-2166GUS strains were significantly lower (~4-fold and ~6-fold lower, respectively) than in WT-2166GUS, but similar to that in mock-treated WT-GUS. These data indicated that *hrpG* and *hrpX* regulate *Aac5_2166* promoter activity (Figure 8B).

We speculated that the secretion of T3Es might be impaired in the ΔhrpG and ΔhrpX mutants. To test this hypothesis, we cloned *Aac5_2166* and its native promoter and inserted them into pBBRNolac-4FLAG to concatenate a C-terminal fusion 4 × FLAG-tag. This construct was transformed into *A. citrulli* Aac5 and Δ*hrpG* to generate WT-2166 and ΔhrpG-2166. No signal was detected in the cell lysates and supernatants of ΔhrpG-2166, while a strong signal was detected for the WT-2166 cell lysates and supernatants (Supplementary Image 3). These results indicated that HrpG regulates the expression of *Aac5_2166* and that *Aac5_2166* could not be synthesized in the *hrpG* mutant.

## Discussion

The mechanisms by which pathogenic bacteria regulate T3Es are critical for successful infection (Tampakaki et al., 2010) and have been well characterized in some plant-pathogenic bacteria. For example, *hrpG* and *hrpX* regulate T3Es in *Xanthomonas* spp. (Xue et al., 2014). Moreover, *hrpG*, as an OmpR family regulator, is typically regulated as a core gene of the T3SS by phosphorylation. OmpR family regulators, as response regulator (RR) proteins, belong to the two-component systems that consist of a histidine kinase and an RR protein, which serve as a basic stimulus-response coupling mechanism to allow organisms to sense and respond to changes in environmental conditions (Tampakaki et al., 2010; Li et al., 2014). The histidine kinase transfers a phosphoryl group to the RR in a reaction catalyzed by the RR. This phosphotransfer to the RR results in the activation of a downstream effector domain that elicits a specific response (Stock et al., 2000; Laub and Goulian, 2007). In the HrpG–HrpX pathway, HrpG activates HrpX, an AraC-type transcriptional activator, to regulate T3E expression. Unfortunately, the mechanisms that regulate the T3SS in *A. citrulli* are poorly understood. However, based on the similarity of the *hrp* clusters of *A. citrulli* and *Xanthomonas* spp. (Tampakaki et al., 2010; Burdman and Walcott, 2012), we hypothesized that a similar mechanism exists in *A. citrulli*. This mechanism likely involves genes homologous to those encoding the core proteins that regulate the T3SS in *Xanthomonas* spp. Genome sequence analyses identified two genes from the AAC00-1 genome (GenBank accession NC_008752) that were homologs of *hrpG* and *hrpX*, designated *Aave_0445* and *Aave_0444*. We cloned the homologs of these genes from *A. citrulli* strain Aac5 (Wang T. et al., 2016).

In the past two decades, researchers have shown that *hrpG* and *hrpX* regulate the expression of T3SS (Wengelnik et al., 1996b; Noël et al., 2001; Huang et al., 2009). However, these molecules also have other functions. Guo et al. (2011) showed that *hrpG* and *hrpX* play global roles in coordinating different virulence traits of *X. axonopodis* pv. *citri*, including traits involved in biofilm formation. Wang T. et al. (2016) showed that in Aac5, the transcription of *hrpE*, a core gene of T3SS, consistently is altered when biofilm formation changes, indicating that biofilm formation may be regulated by T3SS. The results of the current study confirm that *hrpG* and *hrpX* regulate biofilm formation in *A. citrulli* strain Aac5.

During the early stages of infection, plant-pathogenic bacteria colonize the host with the aid of biofilm formation. When *hrpG* and *hrpX* were deleted in *A. citrulli*, we observed significant changes in biofilm formation and virulence. As expected, the *hrpG* and *hrpX* mutants lost the ability to induce an HR in *N. tabacum*, which was confirmed by electrolyte leakage assays. Furthermore, pathogenicity on watermelon seedlings was lost in the mutants. Pathogenic bacteria must overcome the plant's immune systems, including PTI and ETI, to achieve successful infection (Zhang et al., 2007). Physiological markers for PTI typically include ROS generation (Boller and Felix, 2009), and the ETI pathway commonly results in localized, rapid PCD (Stork et al., 2015). The T3SS is essential for inducing a defense response in the host (Boller and Felix, 2009). Bacterial resistance mechanisms involve the secretion of T3Es, which come into contact with host-cell receptors and result in suppression of the host immune system (Dickman and Fluhr, 2013; Jiang et al., 2013). We observed that ΔhrpG and ΔhrpX elicited high-level ROS production, indicating that proteins encoded by these genes affect PTI. Additionally, expression of the putative T3E gene *Aac5_2166*, which encodes a YopJ homolog, was downregulated at the transcriptional level—via alterations in promoter activity—and at the translational level in the *hrpG* and *hrpX* mutants. Combined with the observation that the mutants lost the ability to induce an HR in *N. tabacum*, we concluded that the reduction in virulence in the *hrpG* and *hrpX* mutants was because they could not secrete T3Es and therefore, could not suppress PTI and ETI. This suggests that *hrpG* and *hrpX* play key roles in the regulation of T3Es.

Our findings demonstrate that *A. citrulli* strain Aac5 requires *hrpG* and *hrpX* to regulate virulence factors. *hrpG* and *hrpX* contribute to the expression of the T3SS and are involved in biofilm formation in this strain. Importantly, our findings provide, for the first time, strong evidence that *hrpG* and *hrpX* play key roles in *A. citrulli* Aac5 pathogenicity. Though strain Aac5 belongs to the group II strains of *A. citrulli* (Yan et al., 2013; Wang T. et al., 2016), on the basis of a genome sequence for the group I strain pslb65 reported by Wang et al. (2015) (GenBank accession JYHM00000000), it seems that the T3SS system structures of group I and group II strains are homologous. Moreover, key *A. citrulli* T3Es may be identified in *hrpG* and *hrpX* mutants using transcriptome analysis, which will improve our understanding of the molecular mechanisms of *A. citrulli* pathogenicity.

## Author contributions

XZ and TZ designed the research and wrote the paper. XZ, JY, and LY executed the experiments. XZ, MZ, YY, and WG performed the data analyses. XZ, RW, TZ, and MZ critically reviewed the manuscript. All authors read and approved the final manuscript.

### Conflict of interest statement

The authors declare that the research was conducted in the absence of any commercial or financial relationships that could be construed as a potential conflict of interest.

## Supplementary material

Supplementary Table 1 Primers used in the study.

Supplementary Image 1 The seedling syringe-infiltration assays of *Acidovorax citrulli*. **(A)** Response of watermelon cotyledons to syringe infiltration with *Acidovorax citrulli* wildtype strain Aac5, ΔhrpG and the complemented *hrpG* mutant (ΔhrpG-comp). The experiment was conducted three times. **(B)** Responses of watermelon cotyledons infiltrated with different A. *citrkulli strains* including wild-type strain Aac5, the *hrpX* mutant and its complemented strain ΔhrpX-comp. The experiment was conducted three times.

Supplementary Image 2 Programed cell death assay for *Aac5_2166* gene. The *Aac5_2166* full-length gene sequence with primers was cloned and inserted into 1132 vector carrying the 35S promoter and eGFP tag, and transformed into the GV3101 strain to generate the strain 1132-2166. The strain was cultured in LB liquid medium with Km up to OD_600_ = 0.6 and adjusted the bacteria suspension to OD_600_ = 0.3 with buffer containing 10mM MgCl_2_, 10mM MES, 200uM Acetosyringone by 3200 g centrifugation for 10 min at 25°C conditions. The suspension was inoculated into *N. benthamiana* leaves and the empty vector 1132 and buffer were used as negative controls. The photo was taken after 3 days. The experiment was conducted five times.

Supplementary Image 3 Western blotting showed that effector *Aac5_2166* lost the ability to express the protein in Δ*hrpG* mutant. WT-2166, wild-type strain Aac5 transformed with pBBR2166 carrying 4 × FLAG tag; ΔhrpG-2166, ΔhrpG transformed with pBBR2166 carrying 4 × FLAG tag. These strains were cultured with liquid T3SS induced medium up to OD_600_ = 0.5. To extract intracellular protein, 4 ml of cell suspension was treated with Protease Inhibitor Cocktail (Bimake, Shanghai, China). The lysate was centrifuged at 12,000 × g and 4°C for 3 min, and the cells were resuspended in 200 μL of 4 × Laemmli Sample Buffer (Bio-Rad, Beijing, China) and heated for 10 min. To extract secreted proteins, the bacterial cell supernatants were treated with Protease Inhibitor Cocktail (Bimake), separated by centrifugation (Liu et al., 2017), and the proteins were precipitated with 10% trichloroacetic acid (Sigma, Shanghai, China). The intracellular and secreted protein extracts were analyzed by SDS-PAGE and immunoblotting using specific antibodies. The experiment was conducted three times.
