# Supplementary material for: Involvement of hrpX and hrpG in the Virulence of Acidovorax citrulli Strain Aac5, Causal Agent of Bacterial Fruit Blotch in Cucurbits
Source: Front Microbiol. 2018 Mar 27;9:507. doi: 10.3389/fmicb.2018.00507 (PMC5880930; doi:10.3389/fmicb.2018.00507)
Supplement: Supplementary Table 1 — Primers used in the study. [file Table1.docx]

|  | | **Table S1. Primers used in this study.** | | |
| --- | --- | --- | --- | --- |
| **Primer name** | **Sequence (forward/reverse)** | | **Length** | **Description** |
| hrpG-1F | CTATGACATGATTACGAATTCGAGCGTTTCGCCGGACAT | | 1185 bp | For deleting the *hrpG* gene, located upstream of the *hrpG* gene |
| hrpG-1R | CAGGGTCTCAACTGCCCAGGCGACTCGCATGATTTCCCCA | |  |  |
| hrpG-2F | TGGGGAAATCATGCGAGTCGCCTGGGCAGTTGAGACCCTG | | 1146 bp | For deleting the *hrpG* gene, located downstream of the *hrpG* gene |
| hrpG-2R | CAGGTCGACTCTAGAGGATCCGCGACCTGGAAGAACTGGAGC | |  |  |
| hrpX-1F | CTATGACATGATTACGAATTCGCGACAACATCCTGACCTCC | | 1514 bp | For deleting the *hrpX* gene, located upstream of the *hrpX* gene |
| hrpX-1R | ACAGCTTGAAGGCATTCGCCCGTTGGGAGGAGAGAGAAAG | |  |  |
| hrpX-2F | CTTTCTCTCTCCTCCCAACGGGCGAATGCCTTCAAGCTGT | | 1480 bp | For deleting the *hrpX* gene, located downstream of the *hrpX* gene |
| hrpX-2R | CAGGTCGACTCTAGAGGATCCTGGCCGTCATCGACAACCT | |  |  |
| hrpG-TF | GGGAGGCATTCAAGCCATCT | | 417 bp | For confirming the deletion of the *hrpG* mutant |
| hrpG-TR | AACAGCAGCCAGGCGAGTT | |  |  |
| HBhrpG-F | CGCTCTAGAACTAGTGGATCCATTTCGATCGTCGCGGCC | | 1340 bp | For cloning the *hrpG* full-length ORF carrying native promoter sequence |
| HBhrpG-R | GGTAAGCTTGATATCGAATTCACTGCCCAGGGGCGG | |  |  |
| hrpX-TF | GTCGCACCCTGCTGCTGATAG | | 411 bp | For confirming the deletion of the *hrpX* mutant |
| hrpX-TR | AGGCGTGGTCCGACAGTTCTT | |  |  |
| HBhrpX-F | CGCTCTAGAACTAGTGGATCCATGATTTCCCCATACGCAAAC | | 1946 bp | For cloning the *hrpG* full-length ORF carrying native promoter sequence |
| HBhrpX-R | GGTAAGCTTGATATCGAATTCGTGCCGCATCGACGACAG | |  |  |
| Km-F | ATGATTGAACAAGATGGATTGCAC | | 795 bp | Confirming the absence of Kan resistance fragment in the deletion mutants |
| Km-R | TCAGAAGAACTCGTCAAGAAGGC | |  |  |
| WFB1 | GACCAGCCCACACTGGGAC | | 360 bp | For confirming *Acidovorax citrulli* strains |
| WFB2 | CTGCCGCACTCCAGCGA | |  |  |
| HB2166F | CGCTCTAGAACTAGTGGATCCAGCGGCCTACCAGCTCGCCGGGCAG | | 1619 bp | For cloning the *Aac5_2166* full-length ORF carrying native promoter sequence |
| HB2166R | GGTAAGCTTGATATCGAATTCTTCGATAGCTTTTCTGATTTTTCTC | |  |  |
| 2166GUS-F | CGCGGTGGCGGCCGCTCTAGAAGCGGCCTACCAGCTCG | | 542 bp | For cloning the *Aac5_2166* native promoter sequence |
| 2166GUS-F | CATAAGCTTGATATCGAATTCAAACCATTCTCAATTGCAAA | |  |  |
| GUS-F | CAGGAATTCGATATCAAGCTTATGGTCCGTCCTGTAGAAACCC | | 1854 bp | For cloning the GUS reporter gene ORF |
| GUS-R | GGTACCGGGCCCCCCCTCGAGTCATTGTTTGCCTCCCTGCT | |  |  |
| lac-F | GCCTGGGGTGCCTAATGAGTGAGCT | |  | For deleting the lac promoter |
| lac-R | TGTGGAATTGTGAGCGGATAACAAT | |  |  |
| 4ftag-F | CAGGAATTCGATATCAAGCTTCACCGGCACCAAGACCGA | |  | For inserting the 4×FLAG tag |
| 4ftag-R | GGGCCCCCCCTCGAGGTCGACGCCGCGGGTTCACTTGTC | |  |  |
| 4F-2F | ACCGATTACAAGGATGACGATGACA | |  | For inserting the 4×FLAG tag |
| 4F-2R | AAGCTTGATATCGAATTCCTGCAGC | |  |  |
| RT-hrpG-F | GGGAGGCATTCAAGCCATCT | | 417 bp | For detecting *hrpG* mRNA in q-PCR assay |
| RT-hrpG-F | AACAGCAGCCAGGCGAGTT | |  |  |
| RT-hrpX-F | GTCGCACCCTGCTGCTGATAG | | 411 bp | For detecting the *hrpX* mRNA in q-PCR assay |
| RT-hrpX-F | AGGCGTGGTCCGACAGTTCTT | |  |  |
| RT-2166-F | AGAACTGCTGGAAGTGGAGA | | 570 bp | For detecting the *Aac5_2166* mRNA in q-PCR assay |
| RT-2166-R | CAGGGCGAGATTCAAAGA | |  |  |
| RT-rpoB-F | GCGACAGCGTGCTCAAAGTG | | 104 bp | Reference gene in q-PCR assay |
| RT-rpoB-R | GCCTTCGTTGGTGCGTTTCT | |  |  |
| 1132-2166F | CGCTCTAGAACTAGTGGATCCATGAAGAATTTCATGCGATC | | 1080 bp | For cloning the effector gene *Aac5_2166* |
| 1132-2166R | GGGCCCCCCCTCGAGGTCGACTTCGATAGCTTTTCTGATTTTTC | |  |  |
