# Supplementary figures and images for: Involvement of hrpX and hrpG in the Virulence of Acidovorax citrulli Strain Aac5, Causal Agent of Bacterial Fruit Blotch in Cucurbits
Source: Front Microbiol. 2018 Mar 27;9:507. doi: 10.3389/fmicb.2018.00507 (PMC5880930; doi:10.3389/fmicb.2018.00507)

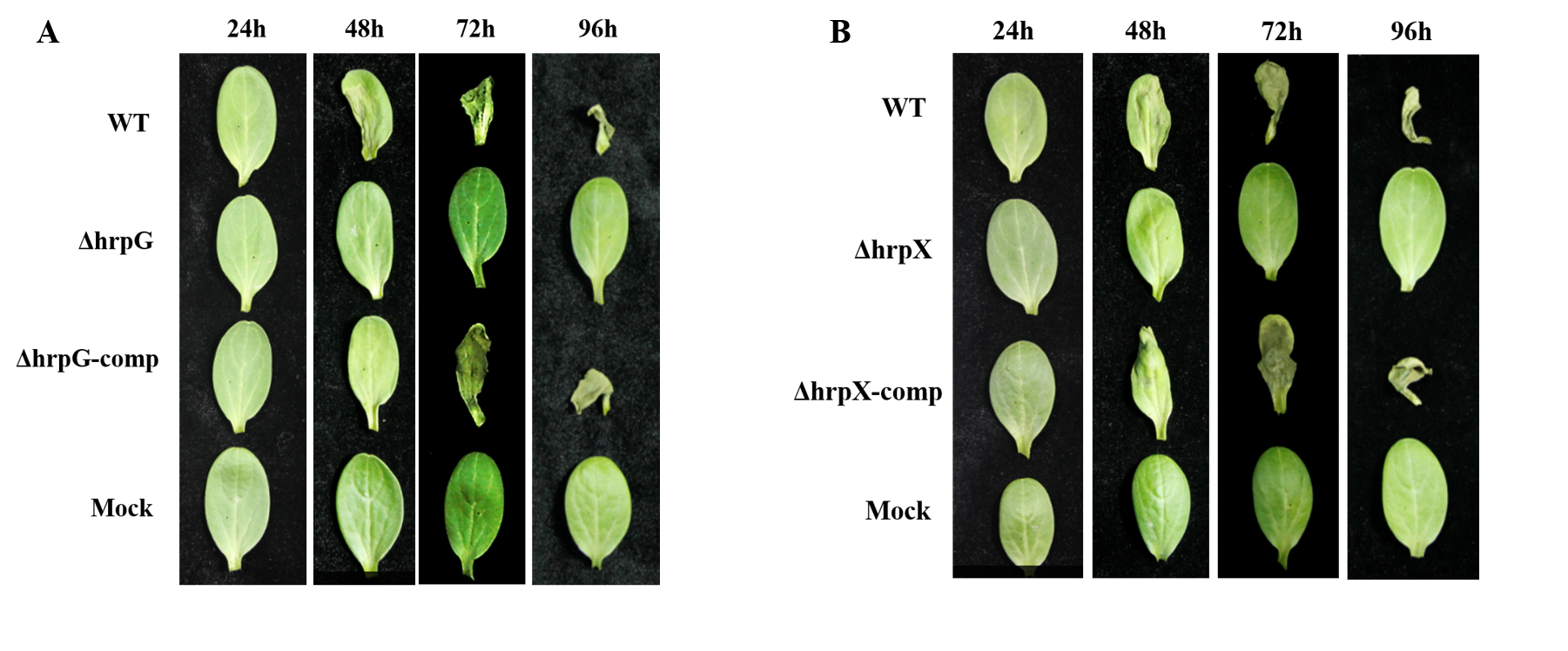

Supplement: Supplementary Image 1 — The seedling syringe-infiltration assays of Acidovorax citrulli. (A) Response of watermelon cotyledons to syringe infiltration with Acidovorax citrulli wildtype strain Aac5, ΔhrpG and the complemented hrpG mutant (ΔhrpG-comp). The experiment was conducted three times. (B) Responses of watermelon cotyledons infiltrated with different A. citrkulli strains including wild-type strain Aac5, the hrpX mutant and its complemented strain ΔhrpX-comp. The experiment was conducted three times. [file Image1.TIF]

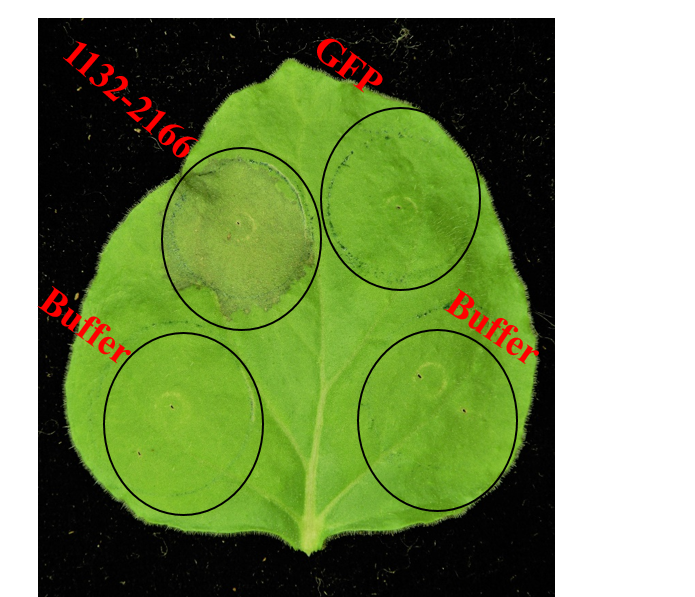

Supplement: Supplementary Image 2 — Programed cell death assay for Aac5_2166 gene. The Aac5_2166 full-length gene sequence with primers was cloned and inserted into 1132 vector carrying the 35S promoter and eGFP tag, and transformed into the GV3101 strain to generate the strain 1132-2166. The strain was cultured in LB liquid medium with Km up to OD600 = 0.6 and adjusted the bacteria suspension to OD600 = 0.3 with buffer containing 10mM MgCl2, 10mM MES, 200uM Acetosyringone by 3200 g centrifugation for 10 min at 25°C conditions. The suspension was inoculated into N. benthamiana leaves and the empty vector 1132 and buffer were used as negative controls. The photo was taken after 3 days. The experiment was conducted five times. [file Image2.TIF]

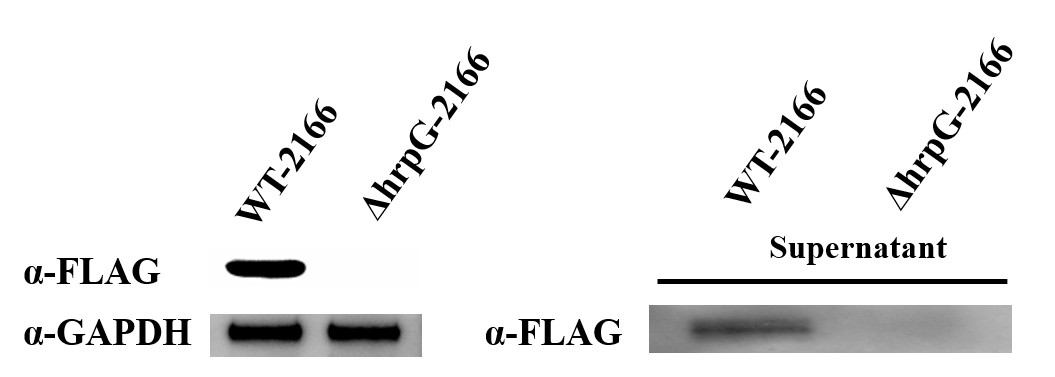

Supplement: Supplementary Image 3 — Western blotting showed that effector Aac5_2166 lost the ability to express the protein in ΔhrpG mutant. WT-2166, wild-type strain Aac5 transformed with pBBR2166 carrying 4 × FLAG tag; ΔhrpG-2166, ΔhrpG transformed with pBBR2166 carrying 4 × FLAG tag. These strains were cultured with liquid T3SS induced medium up to OD600 = 0.5. To extract intracellular protein, 4 ml of cell suspension was treated with Protease Inhibitor Cocktail (Bimake, Shanghai, China). The lysate was centrifuged at 12,000 × g and 4°C for 3 min, and the cells were resuspended in 200 μL of 4 × Laemmli Sample Buffer (Bio-Rad, Beijing, China) and heated for 10 min. To extract secreted proteins, the bacterial cell supernatants were treated with Protease Inhibitor Cocktail (Bimake), separated by centrifugation (Liu et al., 2017), and the proteins were precipitated with 10% trichloroacetic acid (Sigma, Shanghai, China). The intracellular and secreted protein extracts were analyzed by SDS-PAGE and immunoblotting using specific antibodies. The experiment was conducted three times. [file Image3.TIF]
